# Supplementary material for: Microbiota functional activity biosensors for characterizing nutrient metabolism in vivo
Source: eLife. 2021 Mar 8;10:e64478. doi: 10.7554/eLife.64478 (PMC7939548; doi:10.7554/eLife.64478)
Supplement: Figure 2—source data 1. — Plots represent of the log2 fitness score versus log2 fold change in protein abundance for all genes from a given organism: (A) B. thetaiotaomicron VPI-5482, (B) B. vulgatus ATCC 8482, (C) B. ovatus ATCC 8483, and (D) B. cellulosilyticus WH2, under the specified diet condition. Genes from the specified PUL are highlighted in blue. The overrepresentation of genes positioned in the right lower quadrants of the plots (i.e., those showing high expression and low fitness when they are disrupted by a transposon), was defined with a chi-square test using all other genes with both proteomic and INSeq data as the null. The central shaded region represents an ellipse of the inter-quartile range of both the fitness score and protein abundance for that organism under the specified diet condition. This region was excluded from the chi-square calculation of a PUL being overrepresented in the lower right quadrant to increase the stringency of the test. Presented p-values are FDR corrected. [file elife-64478-fig2-data1.pdf]

A

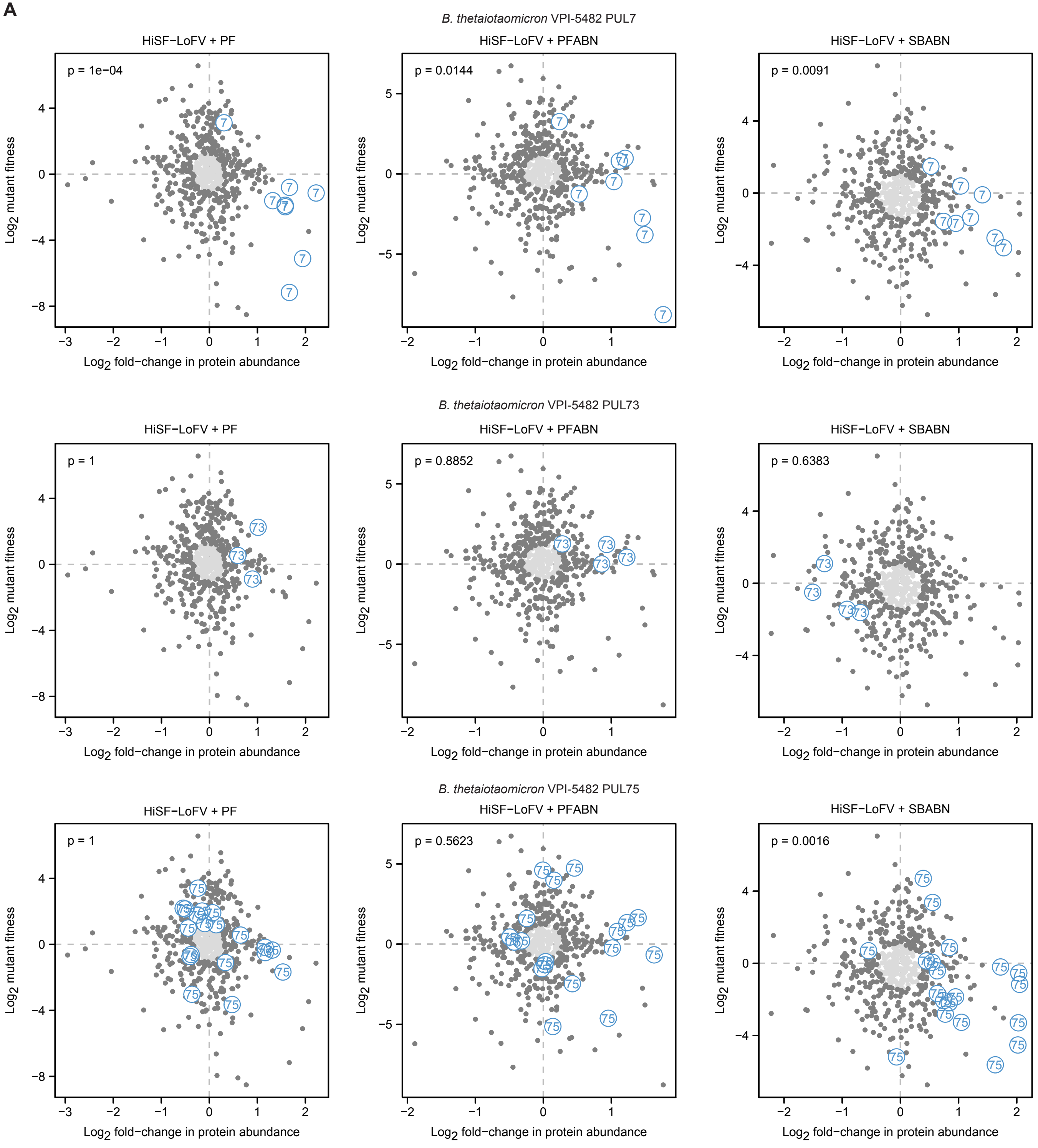
B. thetaiotaomicron VPI-5482 PUL7
HiSF−LoFV + PF
p = 1e−04
HiSF−LoFV + PFABN
p = 0.0144
HiSF−LoFV + SBABN
p = 0.0091
B. thetaiotaomicron VPI-5482 PUL73
HiSF−LoFV + PF
p = 1
HiSF−LoFV + PFABN
p = 0.8852
HiSF−LoFV + SBABN
p = 0.6383
B. thetaiotaomicron VPI-5482 PUL75
HiSF−LoFV + PF
p = 1
HiSF−LoFV + PFABN
p = 0.5623
HiSF−LoFV + SBABN
p = 0.0016
Log2 mutant fitness
Log2 fold−change in protein abundance

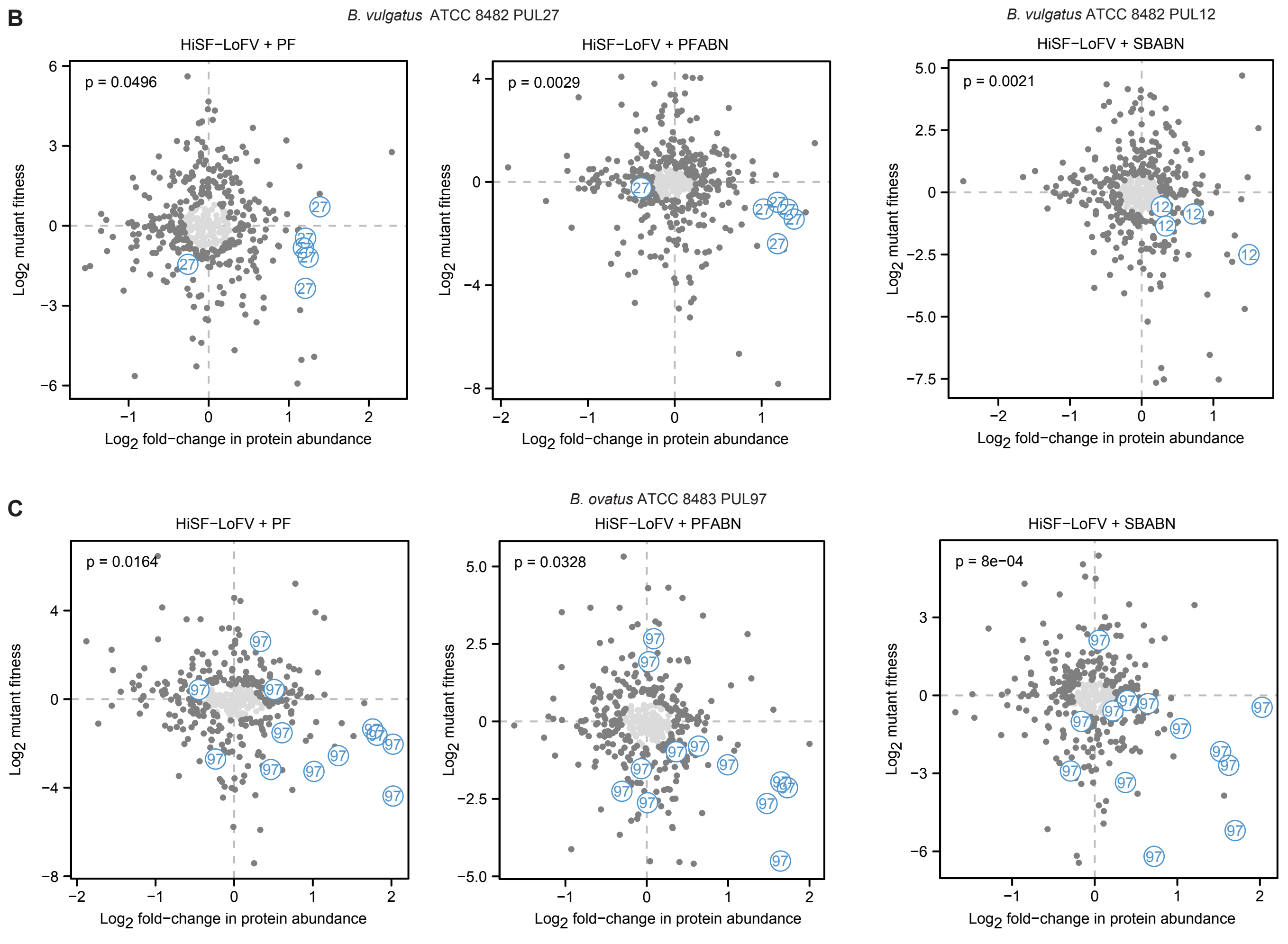

B
B. vulgatus ATCC 8482 PUL27
B. vulgatus ATCC 8482 PUL12
HiSF−LoFV + PF
HiSF−LoFV + PFABN
HiSF−LoFV + SBABN
p = 0.0496
p = 0.0029
p = 0.0021
Log2 mutant fitness
Log2 fold−change in protein abundance
C
B. ovatus ATCC 8483 PUL97
HiSF−LoFV + PF
HiSF−LoFV + PFABN
HiSF−LoFV + SBABN
p = 0.0164
p = 0.0328
p = 8e−04
Log2 mutant fitness
Log2 fold−change in protein abundance

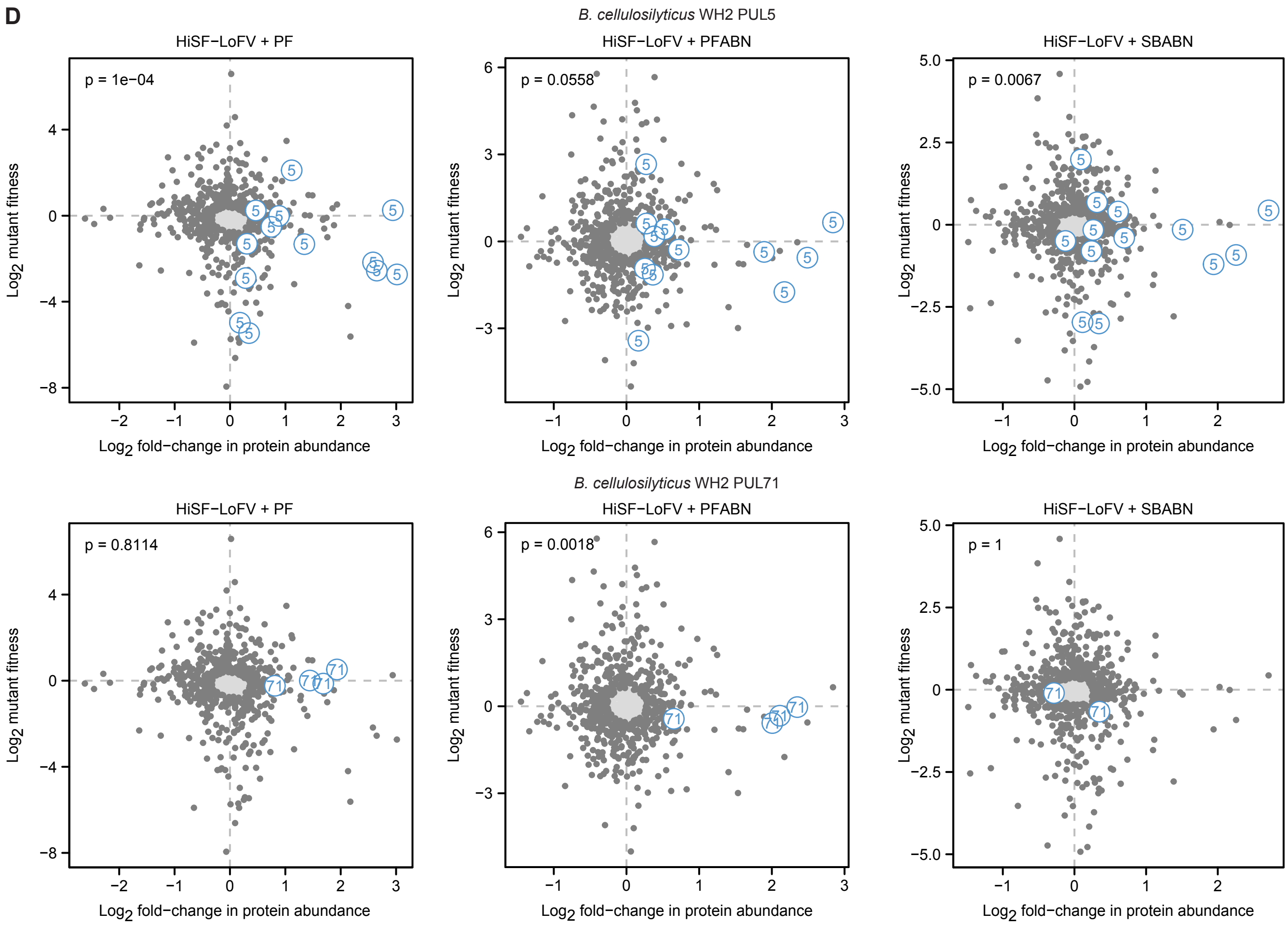
D
B. cellulosilyticus WH2 PUL5
HiSF−LoFV + PF
p = 1e−04
HiSF−LoFV + PFABN
p = 0.0558
HiSF−LoFV + SBABN
p = 0.0067
B. cellulosilyticus WH2 PUL71
HiSF−LoFV + PF
p = 0.8114
HiSF−LoFV + PFABN
p = 0.0018
HiSF−LoFV + SBABN
p = 1
Log2 mutant fitness
Log2 fold−change in protein abundance
